# A brief guide to analyzing TurboID-based proximity labeling-mass spectrometry in plants

**DOI:** 10.1016/j.mocell.2025.100236

**Published:** 2025-06-03

**Authors:** Tae-Ki Park, Tae-Wuk Kim

**Affiliations:** 1 Department of Life Science, Hanyang University, Seoul 04763, Republic of Korea; 2 Hanyang Institute of Bioscience and Biotechnology, Hanyang University, Seoul 04763, Republic of Korea; 3 Research Institute for Convergence of Basic Science, Hanyang University, Seoul 04763, Republic of Korea

**Keywords:** Arabidopsis, Mass spectrometry, Protein interactome, Proximity labeling, TurboID

## Abstract

Proximity labeling-mass spectrometry has emerged as a powerful biological tool for elucidating the intracellular protein interactome of target proteins. Here, we introduce experimental strategies and methodologies for understanding complex protein-protein interactions in plant cells using the recently developed TurboID-based PL approach. We provide a brief overview of the overall procedures, including the preparation of transgenic Arabidopsis plants, biotin treatment, affinity purification, on-bead digestion, liquid chromatography-tandem mass spectrometry, and data analysis.

## INTRODUCTION

Elucidating protein-protein interactions is a fundamental yet essential step in understanding various biological processes and their underlying mechanisms within cells. To achieve this, researchers have employed various experimental techniques. Traditional methods such as yeast 2-hybrid, in vitro pull-down, bimolecular fluorescence complementation, and coimmunoprecipitation (Co-IP) assays have been widely used to determine whether a target protein interacts with a specific binding partner (Bruckner et al., 2009, Lin and Lai, 2017, Miller et al., 2015). On the other hand, for broader exploration of protein interaction networks in cellular environments, Co-IP coupled with mass spectrometry (Co-IP-MS) has been commonly used as an in vivo approach (Free et al., 2009, Lin and Lai, 2017). However, Co-IP-MS has significant limitations. Since Co-IP requires cell lysis, it often leads to high false-positive rates due to nonspecific interactions between proteins originating from different cellular compartments (Dunham et al., 2012, Miernyk and Thelen, 2008, Smirle et al., 2013, Snider et al., 2015). Additionally, Co-IP-MS is ineffective in detecting transient or weak interactions, such as those between enzymes and their substrates (Dunham et al., 2012, Snider et al., 2015, Yang et al., 2021).

Proximity labeling-mass spectrometry (PL-MS) has emerged as a powerful alternative to overcome these limitations. By enabling the identification of protein-protein interactions, protein interactomes, and even protein-nucleic acid interactions within living cells, PL-MS provides a more accurate representation of molecular interactions in their native context (Fazal et al., 2019, Kim et al., 2023, Mair et al., 2019, Myers et al., 2018). This mini-resource describes the application of PL using TurboID, a biotin ligase, in the model plant *Arabidopsis thaliana*, outlining the overall methodology for its use in plant research.

## MAIN BODY

### Overview of PL-MS

PL works by using engineered enzymes that covalently tag nearby proteins with a reactive label (eg, biotin), enabling their selective isolation and identification by MS (Gingras et al., 2019, Kim and Roux, 2016). A peroxidase (eg, APEX) or a biotin ligase (eg, BioID) is genetically fused to a bait protein of interest (Choi-Rhee et al., 2004, Lam et al., 2015, Rhee et al., 2013, Roux et al., 2012). When this fusion protein is expressed in living cells, the enzyme catalyzes the covalent attachment of biotin to proximal proteins within a limited radius (typically 10-20 nm) (Rhee et al., 2013). APEX uses hydrogen peroxide (H₂O₂) to generate short-lived radicals that label tyrosine residues, while BioID uses ATP to activate biotin, which then reacts with lysine residues of proximal proteins. However, APEX has experimental limitations due to cellular toxicity caused by oxidative stress from H₂O₂ and issues with the cellular permeability of peroxidase substrates (Branon et al., 2018, Cho et al., 2020). In contrast, BioID does not pose significant toxicity concerns but has the problem of low catalytic activity, resulting in inefficient labeling (Kim et al., 2016, Roux et al., 2012). TurboID (TbID) was engineered through multiple mutations to overcome BioID's low activity, resulting in enhanced biotin labeling efficiency (Branon et al., 2018).

**Fig. 1:**
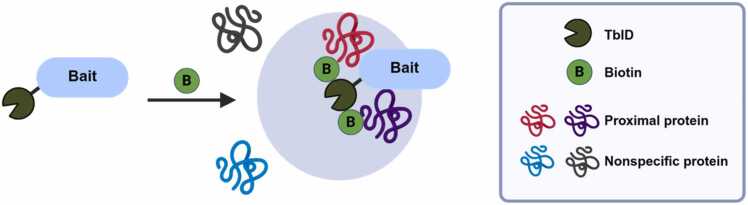
Schematic diagram of TbID-based proximity labeling. A bait-fused TbID (Bait-TbID) protein biotinylates proteins that are located in the proximity of a Bait-TbID in its native cellular environment. Identification of proximal proteins is performed by subsequent affinity purification and mass spectrometry analysis. Schematic drawing produced by BioRender (http://biorender.com/).

**Fig. 2:**
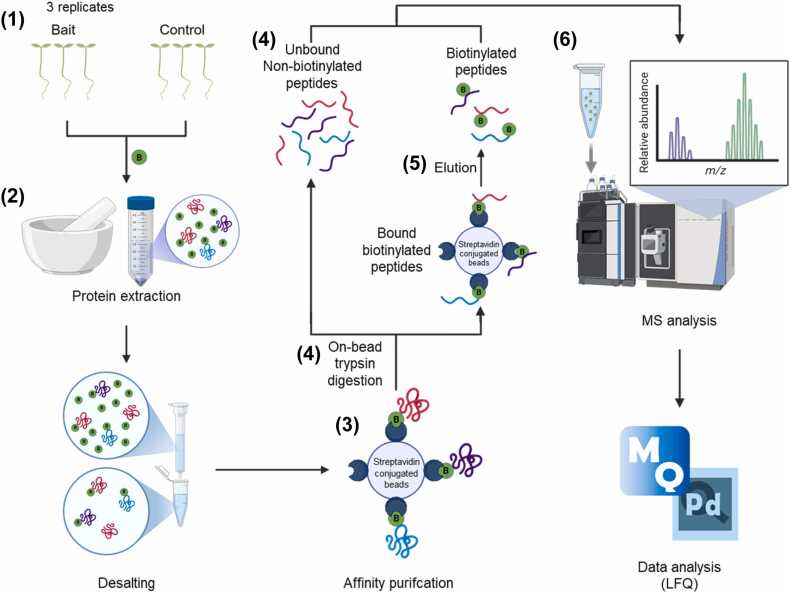
Overview of the step-by-step procedure of TbID-PL-MS. For detailed descriptions of each step, please refer to the manuscript. Schematic drawing produced by BioRender (http://biorender.com/).

To apply the TbID-PL-MS technique in Arabidopsis, a transgenic plant expressing a bait-fused TbID protein (Bait-TbID) must first be generated. When expressed in plant cells, it localizes to the intracellular region corresponding to the bait protein's native environment. In this region, TbID randomly biotinylates lysine residues of interacting or substrate proteins that are in close physical proximity to the bait protein (Fig. 1). After labeling, cells are lysed, and the biotinylated proteins are selectively captured through affinity purification using streptavidin-coated beads, which exhibit exceptional binding affinity for biotin. These enriched proteins undergo enzymatic digestion with trypsin to generate peptide fragments, which are subsequently analyzed via liquid chromatography-tandem mass spectrometry (LC-MS/MS). Finally, protein IDs are identified, allowing for the characterization of the bait protein's interactome (Fig. 2).

### Step 1. Plant Preparation and Biotin Treatment

1. Prepare Arabidopsis plants expressing either the Bait-TbID protein or yellow fluorescent protein (YFP)-fused TbID. Fluorescent protein-fused TbID can serve as a good negative control.
2. Sterilize seeds using 70% ethanol containing 0.025% Triton-X-100 and plant seeds on 0.5× MS medium (Duchefa Biochemie, Haarlem, the Netherlands) supplemented with 0.8% phytoagar and 1% sucrose.
3. After cold stratification for 2 days at 4°C, grow seedlings for 7 to 10 days in a plant growth chamber at 22 to 23°C under a 16 hours light/8 hours dark cycle (light intensity: 80 μmol m⁻² s⁻¹).
4. Harvest seedlings and incubate them in a 50 μM biotin solution for 3 hours.

Note: TbID protein acting as a negative control should be engineered to localize to the same subcellular compartment as the bait protein. If bait is only nuclear, add a nuclear localization signal to YFP-TbID (Mair et al., 2019). If the bait is plasma membrane-associated, modify YFP-TbID by incorporating an N-terminal transmembrane domain or a myristoylation motif for plasma membrane localization (Won et al., 2023). Biotin concentration and incubation time may require optimization through preliminary experiments. To use the Stable Isotope Labeling by Amino Acids in Arabidopsis method for quantitative MS analysis, endogenous proteins must be fully labeled with ¹⁵N, which requires at least 2 weeks of growth (Kim et al., 2023). Therefore, it is not suitable to examine the protein interactome during the early seedling development stage.

Biotin treatment significantly improves the efficiency of PL. However, applying biotin in liquid media may be unsuitable under certain physiological conditions, such as drought stress. Even in these cases, TbID-based PL can proceed without external biotin supplementation, as plants naturally produce biotin, although labeling efficiency may be somewhat reduced.

### Step 2. Protein Extraction and Biotin Desalting

1. Grind 4 g of plant tissues into a fine powder in liquid nitrogen using a mortar and pestle.
2. Extract with 4 mL (1 mL/1 g) of a cold lysis buffer (50 mM Tris pH 7.5, 150 mM NaCl, 0.1% SDS, 1% Triton-X-100, 0.5% SDC, 1 mM EDTA, 10 mM NaF, 1× PI [Pierce Protease inhibitor tablet, #32965], 1 mM PMSF).
3. Sonicate the lysate (Branson, 20% amplitude, 10 seconds on/10 seconds off, for 2 minutes) and centrifuge at 1,500 rpm for 5 minutes to remove debris.
4. Transfer the supernatant and centrifuge again at 12,000 rpm for 10 minutes and collect the final supernatant (∼5 mL) for desalting.
5. Equilibrate a PD-10 desalting column (GE, #17085101) with 25 mL of lysis buffer.
6. Load 2.5 mL of protein extract onto the column and allow it to fully enter the resin, and discard the flow-through during sample loading.
7. Place a new collection tube under the column and elute proteins with 3.5 mL of lysis buffer.
8. Wash the column with 50 mL of lysis buffer (without SDS and SDC) before processing the remaining 2.5 mL of protein extract.
9. Repeat the desalting step for the second portion of protein extract and combine the 2 eluted fractions (total volume: 7 mL).

Note: The desalting step to remove free biotin is crucial. If the treated biotin concentration is low, this step may be omitted. However, at high concentrations, free biotin can interfere with the binding of biotinylated proteins to streptavidin beads, significantly reducing the efficiency of affinity purification (Kim et al., 2023, Mair et al., 2019).

### Step 3. Affinity Purification

1. Incubate the desalted protein extracts with 60 μL Streptavidin magnetic beads (Thermo Scientific, Dynabeads M-280) for several hours or overnight with gentle mixing. Use a magnetic rack to separate extracts solution from beads.
2. Wash beads sequentially with:
  − 1 mL of 50 mM Tris buffer (pH 7.5) with 2% SDS.
  − 1 mL of 50 mM Tris buffer (pH 7.5) with 150 mM NaCl, 0.4% SDS, 1% Triton-X-100, 1.5 mM MgCl_2_, and 1 mM EGTA. Repeat 2 times.
  − 1 mL of 1 M KCl.
  − 1 mL of 0.1 M Na_2_CO_3_.
  − 1 mL 50 mM ammonium bicarbonate solution.
3. Take a small aliquot of the washed beads for immunoblot analysis to confirm biotin labeling.

### Step 4. Elution of Nonbiotinylated Peptides by On-Bead Digestion

1. Add 100 µL of digestion buffer (100 mM Tris-Cl, pH 8.5, 0.5% SDC, and 0.5% SLS) to the microcentrifuge tube containing the washed beads.
2. Add 100 µL of 50 mM triethylammonium bicarbonate containing 1 µg of trypsin. Incubate at 37°C for 4 hours.
3. Place the tube on a magnetic rack to separate the beads from the solution. Transfer the supernatant to a new tube. Keep the beads for use in step 5.
4. Add 50 µL of 10% trifluoroacetic acid, vortex thoroughly, and centrifuge at 12,000 rpm for 20 minutes.
5. Desalt the peptides using a C18 spin column (Thermo Scientific, #89870).
6. Dry the sample using a SpeedVac. Store at −20°C until LC-MS/MS analysis.

Note: (Optional) Prior to trypsin digestion, alkylation using iodoacetamide can be performed after reducing the protein's disulfide bonds to enhance cleavage efficiency.

### Step 5. Elution of Biotinylated Peptides From Streptavidin Beads

1. Wash the streptavidin beads used in step 4 with 200 µL of ddH_2_O 3 times.
2. Add 200 µL of 80% acetonitrile containing 0.2% trifluoroacetic acid and 0.1% formic acid, incubate the beads at 75°C for 5 minutes with gentle shaking (800 rpm), and transfer the solution into a new tube. Repeat 2 more times.
3. Dry the sample using a SpeedVac. Store at −20°C until LC-MS/MS analysis.

### Step 6. LC-MS/MS and Data Analysis

Peptides obtained from steps 4 and 5 are resuspended in 0.1% formic acid and analyzed by LC-MS/MS using a Vanquish Neo System coupled to a Q-Exactive Plus Orbitrap mass spectrometer (Thermo Scientific). The mass spectrometer was operated in the data-dependent acquisition with a full MS scan mode (*m/z* 380-1800). Protein IDs are obtained using Proteome Discoverer or MaxQuant software. From a list of proteins consistently identified in at least 3 independent PL-MS experiments, the bait protein interactome is constructed by selecting (1) proteins detected exclusively in the bait-TbID dataset but not in the YFP-TbID dataset and (2) proteins detected at a frequency higher (eg, 3- or 5-fold) in bait-TbID than in YFP-TbID through label-free quantification. We recommend applying a higher threshold than typically used in transcriptome analyses such as RNA-Seq, as label-free quantification in mass spectrometry has relatively lower quantitative accuracy and is susceptible to missing values for low-abundance proteins, which can reduce data reliability and reproducibility.

## CONCLUDING REMARKS

PL-MS serves as a versatile tool for identifying novel interactions in their native cellular environment and detecting transient interactions such as those between enzymes and substrates. The spatiotemporal-specific protein interaction network of the bait protein can be elucidated through the time-specific expression of Bait-TbID using various promoters or through spatially specific expression targeting specific subcellular organelles (Mair et al., 2019, Schopp et al., 2017). Unlike animals, plants encode biotin biosynthetic genes and naturally synthesize biotin (Patton et al., 1998), suggesting that nonspecific labeling by TbID may occur more readily in plant cells than in animal cells. Given that the knockout mutant for biotin synthase is embryo-lethal (Patton et al., 1998, Schneider et al., 1989), employing transgenic plants expressing Bait-TbID proteins in a knockdown mutant background could significantly improve the accuracy and specificity of proximity labeling upon biotin treatment. In addition, proteins identified as proximal candidates should be validated through additional methods such as yeast 2-hybrid, BiFC, or in vitro pull-down assays. Recently, blue light-activated LOV-TbID has been developed, enabling more precise and sophisticated PL (Lee et al., 2023). With its versatility and high reproducibility, TbID-PL-MS is expected to be increasingly utilized across various applications.

## FUNDING AND SUPPORT

This work was supported by the National Research Foundation of Korea grant funded by the Korean government (Ministry of Science and ICT or Ministry of Education) (2021R1A2C1006617 and RS-2024-00407469 to T.-W.K.). This research was also supported by the Korea Basic Science Institute (National Research Facilities and Equipment Center) grant funded by the Ministry of Education (2023R1A6C101A009 to T.-K.P.).

## CRediT authorship contribution statement

**Tae-Ki Park:** Writing – review & editing, Writing – original draft, Visualization, Resources, Methodology, Formal analysis, Data curation, Conceptualization. **Tae-Wuk Kim:** Writing – review & editing, Writing – original draft, Visualization, Supervision, Resources, Methodology, Funding acquisition, Formal analysis, Data curation, Conceptualization.

## DECLARATION OF COMPETING INTERESTS

The authors declare that they have no known competing financial interests or personal relationships that could have appeared to influence the work reported in this paper. The author Tae-Wuk Kim is an Editor for Molecules and Cells and was not involved in the editorial review or the decision to publish this article.
